# Oxygen anomaly in near surface carbon dioxide reveals deep stratospheric intrusion

**DOI:** 10.1038/srep11352

**Published:** 2015-06-17

**Authors:** Mao-Chang Liang, Sasadhar Mahata

**Affiliations:** 1 Research Center for Environmental Changes, Academia Sinica, Taipei, Taiwan; 2 Graduate Institute of Astronomy, National Central University, Jhongli, Taiwan; 3 Department of Physics, University of Houston, Houston, TX, USA; 4 Institute of Astronomy and Astrophysics, Academia Sinica, Taipei, Taiwan

## Abstract

Stratosphere-troposphere exchange could be enhanced by tropopause folding, linked to variability in the subtropical jet stream. Relevant to tropospheric biogeochemistry is irreversible transport from the stratosphere, associated with deep intrusions. Here, oxygen anomalies in near surface air CO_2_ are used to study the irreversible transport from the stratosphere, where the triple oxygen isotopes of CO_2_ are distinct from those originating from the Earth’s surface. We show that the oxygen anomaly in CO_2_ is observable at sea level and the magnitude of the signal increases during the course of our sampling period (September 2013-February 2014), concordant with the strengthening of the subtropical jet system and the East Asia winter monsoon. The trend of the anomaly is found to be 0.1‰/month (R^2^ = 0.6) during the jet development period in October. Implications for utilizing the oxygen anomaly in CO_2_ for CO_2_ biogeochemical cycle study and stratospheric intrusion flux at the surface are discussed.

Transport of mass and chemical species via the large-scale Brewer-Dobson circulation and synoptic/small-scale mixing from the stratosphere to the troposphere and vice versa^1^,^2^,^3^,^4^,^5^,^6^,^7^ has a significant impact on the oxidation capacity of the troposphere^8^,^9^,^10^ and the radiation budget in the stratosphere^11^. In the Pacific and Atlantic regions, the stratosphere-troposphere exchange occurs predominantly over storm tracks during winter, spring, and fall^12^,^13^,^14^,^15^,^16^,^17^,^18^,^19^; Taiwan is located in such a region. In summer the exchange maximizes its amplitude over the central Asia, supported by various observations made at the Waliguan Observatory (36°17´ N, 100°54´ E, 3816 m, China; NOAA ESRL code: WLG) located on the Tibetan Plateau^20^,^21^,^22^,^23^,^24^,^25^,^26^. In the mid-latitudes of East Asia, ozonesonde and surface observations show a distinct spring maximum (e.g., see ref. 27 and references contained therein). This finding is consistent with the fact that the stratospheric intrusions bringing O_3_ into the troposphere reach their seasonal maximum in the summer^27^,^28^. This is primarily due to unusually strong winds associated with the polar and subtropical jet streams over the east Asian coast and the persistence of cyclogenesis over the western Pacific, resulting in frequent tropopause folding and thus significant intrusion of O_3_ into the troposphere^10^,^25^,^29^,^30^,^31^,^32^. The air remaining in the fold of the upper troposphere moves towards the jet exit regions and re-enters the lower stratosphere. However, stretching stratospheric intrusions to smaller scales, some of which go deeper into the troposphere, leads to irreversible transport^1^,^10^,^33^,^34^,^35^,^36^,^37^. Such deep intrusions of stratospheric air down to the lower troposphere or even to the surface are relevant to tropospheric chemistry^8^,^9^,^10^. Ozone, however, is reactive and with numerous sources at the ground level, which makes the use of ozone for the irreversible branch rather ambiguous. In this work, we analyze the so-called “oxygen anomaly” in CO_2_, a species that has distinct anomaly originating in the middle atmosphere, to study how the changing meteorology affects the cross-tropopause transport. The anomaly in CO_2_ is potentially also a powerful tracer for improving our understanding of the carbon cycle^38^. One advantage of using CO_2_ is that this species is inert in the free troposphere; the isotopic composition of CO_2_ can be modified at the surface and in the middle and upper atmospheres only.

In typical biogenic/atmospheric processes, the partitioning between species’ oxygen-containing isotopologues follows a mass-dependent line, i.e.,


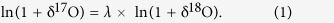


The factor λ is taken to be 0.516 (λ_0_) and may vary between 0.500 and 0.529 (ref. 39). The λ_0_ is chosen following the fractionation that occurs in transpiration at the globally averaged relative humidity of 75% (ref. 40). It has been discovered that some atmospheric species follow a very different relation. For example, δ^17^O(O_3_) ≈ δ^18^O(O_3_) (ref. 41, 42, 43) and δ^17^O(CO_2_) ≈ 1.7 × δ^18^O(CO_2_) in the stratosphere (reference to tropospheric CO_2_ ; ref. 44, 45, 46, 47, 48). The oxygen isotope distribution in CO_2_ is largely affected by O_2_-O_3_-CO_2_ photochemistry in the middle atmosphere, via the reaction O(^1^D) + CO_2_ where O(^1^D) is formed by dissociation of O_3_ (ref. 45,46,48, 49, 50). As O_3_ and CO_2_ are strongly coupled in the stratosphere and isotopically anomalous CO_2_ can be produced in the middle atmosphere only, one may obtain a better constraint for stratospheric O_3_ at the surface by measuring the isotopic composition of CO_2_. Symbol Δ is frequently used to quantify the deviation from the mass-dependent fractionation line, and is defined by


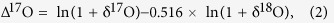


where δ-values are expressed relative to V-SMOW.

## Methods

CO_2_-O_2_ oxygen isotope exchange method developed previously^51^ was followed with slight modification (see Figure S1) to measure the Δ^17^O of CO_2_ samples. The exchange was carried out in a reaction tube (made of quartz, 60 cm in length and 6.5 mm in diameter) with a cold finger and positioned horizontally inside a cylindrical heater. The heating zone is about 15 cm. Isotopic analyses were done using a FINNIGAN MAT 253 mass spectrometer in dual inlet mode. The analytical precision obtained for Δ^17^O values of CO_2_ is 0.008‰ (1-σ standard deviation and hereafter, unless otherwise stated; see Table S1). The precision is also verified by analysis of duplicate samples with difference between duplicates less than 0.01‰. To establish the accuracy of the present method, we follow a typical method^52^ to convert isotopically known O_2_ to CO_2_ and assume the conservation of Δ^17^O. Good accuracy is demonstrated in Table S2 and Figure S2.

Concentration of CO_2_ is measured with a LI-COR infrared gas analyzer (model 840 A, LI-COR, USA) at 4 Hz, smoothed with 20-s moving average. The reproducibility is better than 1 ppmv. The analyzer is calibrated against a compressed air cylinder, with calibrated concentration of 387.7 ppmv. This working standard is calibrated using a commercial Picarro analyzer (model G1301, Picarro, USA) by a series of NOAA/GMD certified tertiary standards with CO_2_ mixing ratios of 369.9, 392.0, 409.2, and 516.3 ppmv. The precision (1-σ) is better than 0.2 ppmv.

## Air sampling

Air samples were collected between September 2013 and February 2014 in cleaned pre-conditioned 1-liter pyrex bottles. The cleaning was done by passing of dry high purity nitrogen overnight. Sampling bottles used for concentration measurements (~350-ml bottle) and bottles used for isotope analyses were connected in series. The sampling was carried out at Academia Sinica campus (abbreviated AS; 121°36'51'' E, 25°02'27'' N; ~10 m above the ground level or 60 m above sea level) in Taipei, Taiwan and the campus of National Taiwan University (NTU; 121°32'21'' E, 25°00'53'' N; ~10 m above the ground level or 20 m above sea level; ~10 km southwest of Academia Sinica). Sampling was done after flushing the bottles for 5 minutes by pumping air at a flow rate of ~2 liter per min. Moisture was removed during sampling by using magnesium perchlorate, to minimize subsequent isotope exchange between CO_2_ and water; the use of magnesium perchlorate reduced moisture content from the ambient value of 70–90% to less than 1% relative humidity, checked using the LI-COR infrared gas analyzer (model 840 A, LI-COR, USA). To get 2-liter equivalent air, we compressed the gas in the bottle to 2 bar. This allows us to get sufficient CO_2_ for isotope analysis (~30 μmole). In addition to major gases like N_2_, O_2_, and Ar, the flask air samples with CO_2_ also contain traces of water vapor and other gases that could potentially interfere with the CO_2_ isotope analysis. Water vapor and a few other condensable gases were removed cryogenically while pumping away the major gases using a glass vacuum system with five traps (a slight modification of ref. 53). Two traps were used at dry ice temperature (−77 ^o^C) for removing water and volatile organics while the remaining three were used for CO_2_ collection at liquid nitrogen temperature (−196 ^o^C). The flow rate was maintained at 100 ml/min during the pumping at a pressure of about 10 to 15 torr. The above process was checked by several control experiments to ensure that there is no escape of CO_2_ and attendant isotope fractionation.

## Results

In general, about 3 samples per day were collected and analyzed, summing up to a total of 81 samples. This is the largest set of data after Thiemens *et al*.^54^ decadal record. In this paper, we focus on the changes in monthly scale and the data are averaged diurnally. The diurnally averaging is applied to minimize diurnal variation due to photosynthesis and respiration. Table 1 summarizes the results from the mission. On average, the concentration ([CO_2_]) is 411.8 ± 9.8 ppmv, δ^13^C −8.91 ± 0.56‰ (V-PDB), δ^18^O 40.60 ± 0.52‰ (V-SMOW), and Δ^17^O 0.329 ± 0.037‰ (1-σ standard deviation to represent the scatter of the data). Identification of the sources responsible for the changes of CO_2_ level can be done from the so-called Keeling plot (Figure S3). The intercept for δ^13^C is −27‰, a value that is consistent with respiration from C_3_ plants (major type of plant in the region), though the signature may not be distinguishable from fossil fuel burning^55^.

**Figure 1:**
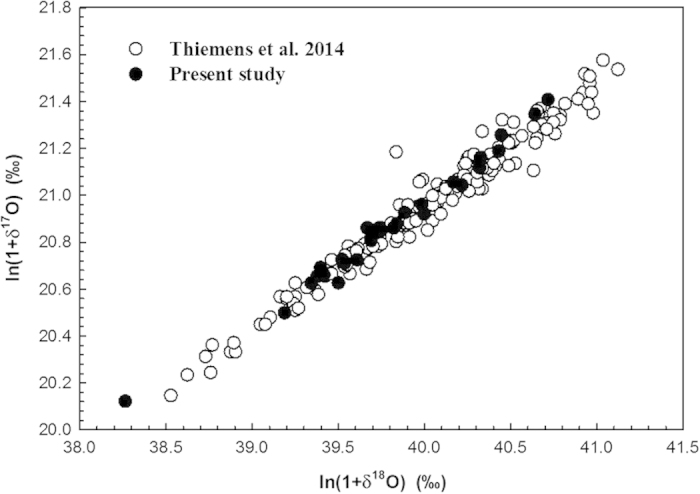
Triple isotope plot of oxygen for CO_2_. The recently published tropospheric data^41^ is also shown for comparison. Values are referenced to V-SMOW.

**Figure 2:**
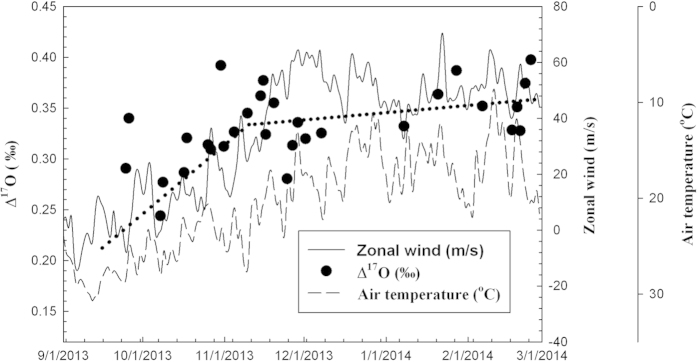
Time series of Δ^17^O obtained during the study period. To show how meteorology and large scale transport affect the Δ^17^O measured at the surface, surface air temperature (taken from the Central Weather Bureau, Taiwan; station code: 466920) and 200-mbar zonal wind (taken from ECMWF Interim reanalysis) are over plotted. Note that the scale of temperature is reversed, for better comparison with the zonal wind. Linear least-square fits to Δ^17^O are shown by the dotted lines (see text).

Figure 1 shows the three-isotope plot of oxygen in CO_2_ collected in the region, comparing to that at La Jolla^54^. Overall, our values agree with Thiemens *et al*.’s. The linear least-square fitting to our data yields a slope of 0.525 ± 0.013 and the value is 0.503 ± 0.008 for Thiemens *et al*.’s. Figure 2 compares the oxygen anomaly with the 200-mbar zonal wind, a proxy for the strength of the subtropical jet. Before Oct, the zonal wind is small and fluctuates around zero. As of then, the westerly is established and the Δ^17^O follows. The average Δ^17^O values for Sep-Oct (2013), Nov-Dec (2013), and Jan-Feb (2014) are 0.309, 0.325, and 0.357‰, respectively. During the jet development period in Oct, the Δ^17^O trend is 0.0035‰/day (R^2^ = 0.59; Fig. 2). Afterwards, further strengthening of the jet does not enhance Δ^17^O and the trend reduces to 0.0004‰/day (R^2^ = 0.22), but the short-term enhancement is apparent (see below).

## Discussion and summary

Stratospheric intrusions in East Asia occurs in close association with the presence of the subtropical jet stream^25^,^26^,^27^,^28^,^29^,^30^,^31^. The jet is situated at ~40 °N in summer and moves southward to Taiwan at ~25 °N in winter. Summer and winter monsoons are two major climate systems responsible for the seasonal changes in Taiwan. The air mass originating from the Asian continent flows through the Pacific Ocean to the island in fall, winter, and spring. Convective activities occurring during the northeast monsoon and accompanied by the passage of mid-latitude cold fronts are largely responsible for the changing meteorology in fall. Cold surges with an abrupt change in temperature are associated with a strong northeasterly wind in winter, followed by cold fronts in spring. Such changing meteorology is also reflected in the subtropical jet stream. The correlation is clearly seen from Fig. 2 that the 200-mbar zonal wind (a proxy for subtropical jet) follows the surface air temperature; in general, the strengthening of winter monsoon (indicated by temperature decrease) is closely associated with the elevated zonal wind speed. This variable meteorology that affects the transport and mixing at all scales can potentially enhance vertical transport into the troposphere^56^ and sometimes also into the upper troposphere and lower stratosphere, thus leading to an enhancement of cross-tropopause exchange resulting in elevated Δ^17^O in surface CO_2_. Below we focus our discussion on the downwelling branch of transport to the lower troposphere.

**Figure 3:**
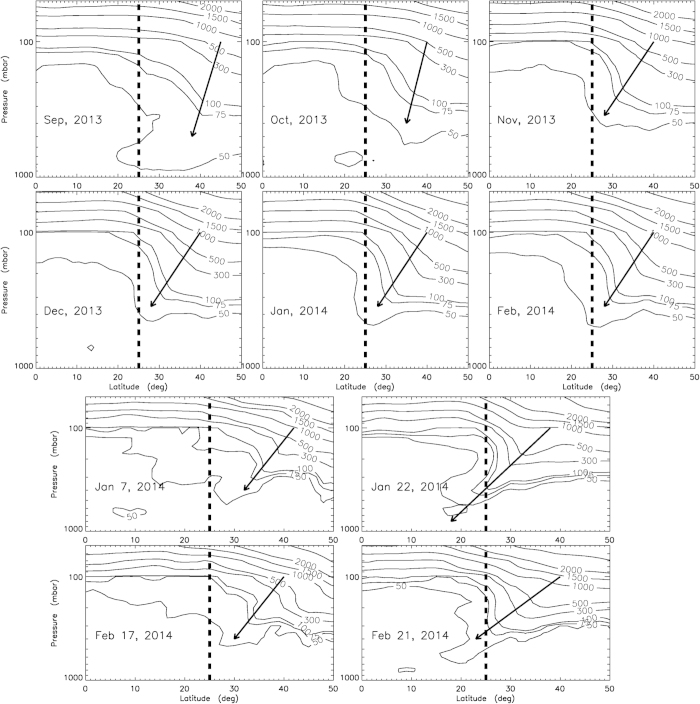
Profiles of ozone volume mixing ratios (ppbv; taken from ECMWF Interim reanalysis) at altitudes from 50 to 1000 mbar pressure level and latitudes from 0 to 50 °N at longitude 121.5 °E. Data are either monthly (top six panels) or diurnally (bottom four panels) averaged. Arrows indicate the movement of stratospheric air toward our sampling site shown by the vertical dashed line.

To support the stratospheric origin of anomalous CO_2_, we analyze ECMWF Interim O_3_ data and the results are presented in Fig. 3. We see that the level of O_3_ at ~200 mbar increases from Sep, 2013 through Feb, 2014. Moreover, stratospheric air moves clearly towards our sampling site (shown by arrows). To further demonstrate the correlation of the intrusion of stratospheric air and the surface CO_2_ oxygen anomaly, two events in 2014 are selected: Jan 07-27 and Feb 17-24. Δ^17^O increases with time, concordant with the elevated zonal wind (Fig. 2; with lag of a few days); the Δ^17^O value changes from 0.332 to 0.387‰ for the former case and from 0.328 to 0.397‰ for the latter. During this time, a large stratospheric intrusion is seen on Jan 22 and Feb 21. The strength of this intrusion is much stronger than that on Jan 07 and Feb 17, respectively (see Fig. 3). The trend of Δ^17^O is calculated to be 0.0098‰/day (R^2^ = 0.79) for the latter case and is a factor of ~3 higher than the former (0.0027‰/day; R^2^ = 0.99) and the trend in Oct (0.0035‰/day; R^2^ = 0.59).

The isotopic composition of CO_2_ in the atmosphere is an integrated signal of atmospheric and biogeochemical processes. In the atmosphere, the primary mechanism that modifies the isotopic composition of CO_2_ is the exchange reaction with O(^1^D) in the stratosphere. The stratospheric source of CO_2_ is enhanced in δ^18^O and Δ^17^O and has a seasonal cycle that is different from that originating from the surface^21^,^57^. For example, at the Waliguan observatory, biogeochemical models^21^ predict maximum effects due to respiration in March-April (maximum in δ^18^O) and August (minimum in δ^18^O) and due to assimilation in ~March (minimum in δ^18^O) and July-August (maximum in δ^18^O), while the Brewer-Dobson circulation has maximum strength in ~March-July^36^. As a consequence of the interaction between these processes, the maximum in δ^18^O may occur in June^57^. This does not mean the seasonal cycle of δ^18^O is solely caused by the cross-tropopause exchange [cf. ref. 58]. Instead, in addition to natural biogeochemical cycle that results in maximal δ^18^O in ~April, elevated δ^18^O from the stratosphere is to modify the seasonal cycle to move the peak from April to June^57^. The presence of frequent deep intrusions over Tibet was shown recently^28^. However, to fully resolve the source of summertime O_3_, tracers like Δ^17^O that are seriously affected by stratospheric processes are essential. In this work, the size of Δ^17^O elevated during the subtropical jet strengthening period is up to ~0.1‰ (trend of 0.0035‰/day over one-month in Oct), a value that is expected by bringing air with 1‰ (referenced to the mean anomaly of tropospheric CO_2_) anomaly^45^,^59^ from ~100 mbar to 1000 mbar. Attempts to utilize Δ^17^O for stratospheric and surface flux estimates are made below.

Given that CO_2_ is chemically inert in the troposphere, assuming steady state, we have


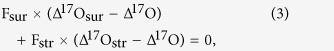


where F_sur_ and F_str_ are the fluxes from the surface and stratosphere, respectively. Δ^17^O_sur_ and Δ^17^O_str_ are the corresponding oxygen anomalies. F_sur_ includes the CO_2_ fluxes associated with photosynthesis, respiration, soil invasion, and oceanic processes. Assuming the globally averaged surface emitted CO_2_ is in isotopic equilibrium with water at 25 °C, δ^18^O = 41‰ and Δ^17^O_sur_ = (0.523–0.516) × ln(1 + δ^18^O) = 0.281‰, where 0.523 is the equilibrium constant of water and CO_2_ (ref. 60) and 0.516 is our adopted slope (following equation 2). If we take 0.522 equilibrium value^61^, Δ^17^O_sur_ reduces to 0.241‰, providing a likely explanation to the obtained low Δ^17^O on Oct 07. The low value can also be of anthropogenic origin, as combustion produces Δ^17^O as low as about −0.2‰ (ref. 62), and this is supported by the elevated [CO_2_] and reduced δ^13^C on that day (see Table 1).

**Figure 4:**
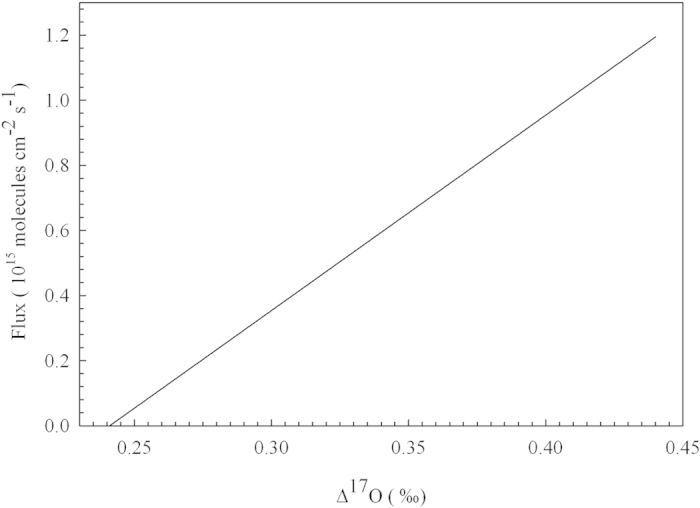
Inferred flux from the stratosphere obtained by assuming F_sur_ = 3 × 10^15^ molecules cm^−2^ s^−1^, Δ^17^O_sur_ = 0.241‰, and Δ^17^O_str_ - Δ^17^O = 0.5‰ (see text).

Taking daytime photosynthetic flux of 10^15^ molecules cm^−2^ s^−1^ from a direct flux measurement for CO_2_ in a subtropical forest^63^ and following the same assumption as *Hoag et al*.^38^ for C_3_ plants, we have F_sur_ = 3 × 10^15^ molecules cm^−2^ s^−1^. (Δ^17^O_str_ - Δ^17^O) is 0.5–1‰ (ref. 45,59). F_str_ can then be evaluated from equation (3). Figure 4 shows the estimated flux from the stratosphere, providing a way to assess the vertical transport in transport models in the stratosphere, troposphere, and boundary mixed layer. We note that F_sur_ remains poorly understood. Hence similarly, if one can get an improved understanding for F_str_ from, for example, extensive mid-tropospheric measurements^58^, F_sur_ can be better determined. We expect the utilization of multiple tracers (such as N_2_O) obtained by the CARIBIC project^58^ along with Δ^17^O in CO_2_ and a global model^45^,^46^,^64^ could place a strong constraint on the strength of the cross-tropopause exchange, in particular with the use of the correlation of Δ^17^O and [N_2_O] in the upper troposphere. The uniqueness of Δ^17^O and [N_2_O] lies on their chemical properties in the atmosphere: both are inert in the free troposphere but significantly altered in the stratosphere^45^,^47^,^59^,^64^.

In short, stratosphere-troposphere exchange carries stratospheric air to the troposphere. The air from the stratosphere has oxygen isotope signature of CO_2_ distinct from that originating from the surface. The interaction between the subtropical jet and winter monsoon systems could enhance the vertical mixing and cross-tropopause exchange, supported by the observed Δ^17^O in the near surface air CO_2_. The detection of Δ^17^O trend is clearly demonstrated. The magnitude of the trend is found to be correlated with the strengths of the subtropical jet and winter monsoon. This trend is, on average, 0.0035‰/day during the jet development period in Oct, and can be as much as 0.0098‰/day that we observe in Feb. The observed anomalous CO_2_ at the surface potentially provides an additional constraint to refine our view of carbon cycle involving CO_2_ and also provides a strong constraint on the transport of the stratospheric flux to the surface. This is the largest dataset after Thiemens *et al*.^54^ and the first attempt to monitor Δ^17^O at such a high sampling frequency.

## Additional Information

**How to cite this article**: Liang, M.-C. and Mahata, S. Oxygen anomaly in near surface carbon dioxide reveals deep stratospheric intrusion. *Sci. Rep*. **5**, 11352; doi: 10.1038/srep11352 (2015).

## Supplementary Material

Supplementary Information

## Figures and Tables

**Table 1:** Summary of CO_2_ data collected at the campus of Academia Sinica and National Taiwan University. Values of δ^13^C and δ^18^O are referenced to V-PDB and V-SMOW, respectively. The error bar represents 1-σ standard deviation (scatter) of diurnal data. Isobaric interferences of N_2_O to δ^13^C and δ^18^O have been corrected.

Sampling date (number of samples)	[CO_2_] (ppmv)	δ^13^C (‰)	δ^18^O (‰)	Δ^17^O (‰)
Academia Sinica				
2013/09/24 (3)	397.9 ± 9.3	−8.35 ± 0.45	41.04 ± 0.30	0.291 ± 0.033
2013/09/25 (2)	400.5 ± 2.9	−8.50 ± 0.16	40.54 ± 0.59	0.340 ± 0.017
2013/10/07 (3)	421.5 ± 35.9	−9.32 ± 1.53	40.29 ± 0.95	0.244 ± 0.025
2013/10/08 (3)	427.4 ± 4.6	−9.61 ± 0.19	39.97 ± 0.32	0.277 ± 0.028
2013/10/16 (3)	409.4 ± 9.5	−8.89 ± 0.41	40.40 ± 0.15	0.287 ± 0.016
2013/10/17 (5)	409.6 ± 14.4	−8.63 ± 0.67	40.20 ± 0.40	0.321 ± 0.011
2013/10/25 (3)	412.4 ± 2.4	−8.86 ± 0.12	40.33 ± 0.16	0.314 ± 0.021
2013/10/26 (2)	420.6 ± 14.2	−9.24 ± 0.55	40.33 ± 0.10	0.310 ± 0.004
a2013/10/30 (2)	394.6	−7.15 ± 0.27	40.46 ± 0.07	0.392 ± 0.033
2013/10/31 (3)	401.0 ± 4.4	−8.48 ± 0.19	41.14 ± 0.11	0.312 ± 0.028
2013/11/04 (3)	410.6 ± 4.5	−8.81 ± 0.19	40.49 ± 0.12	0.327 ± 0.019
2013/11/09 (2)	415.2 ± 14.2	−8.89 ± 0.78	40.69 ± 0.55	0.345 ± 0.015
2013/11/19 (3)	417.5 ± 2.6	−8.79 ± 0.11	40.54 ± 0.04	0.355 ± 0.017
2013/11/26 (3)	408.1 ± 2.2	−8.87 ± 0.64	40.62 ± 0.31	0.313 ± 0.023
2014/01/27 (2)	402.5 ± 2.3	−8.61 ± 0.08	41.27 ± 0.06	0.387 ± 0.004
2014/02/03 (5)	416.3 ± 16.5	−9.17 ± 0.64	41.15 ± 0.44	0.352 ± 0.035
2014/02/17 (3)	430.5 ± 19.5	−9.65 ± 0.81	40.99 ± 0.57	0.328 ± 0.033
2014/02/19 (2)	421.0 ± 4.2	−9.25 ± 0.19	40.49 ± 0.14	0.351 ± 0.032
2014/02/22 (2)	401.5 ± 0.7	−8.40 ± 0.06	41.48 ± 0.02	0.374 ± 0.036
2014/02/20 (2)	413.9 ± 4.7	−8.91 ± 0.15	40.80 ± 0.20	0.328 ± 0.038
2014/02/24 (1)	406.6	−8.63	41.56	0.397
National Taiwan University				
2013/11/14 (3)	394.0 ± 59.3	−8.64 ± 1.13	40.18 ± 1.62	0.362 ± 0.011
2013/11/15 (3)	425.2 ± 11.6	−9.41 ± 0.44	39.01 ± 0.64	0.377 ± 0.039
2013/11/16 (2)	410.3 ± 2.7	−8.74 ± 0.06	40.13 ± 0.16	0.324 ± 0.000
2013/11/24 (3)	413.0 ± 56.3	−9.25 ± 0.56	40.81 ± 0.92	0.281 ± 0.057
2013/11/28 (3)	418.9 ± 3.6	−9.36 ± 0.22	40.16 ± 0.11	0.336 ± 0.056
2013/12/01 (3)	409.9 ± 2.4	−8.89 ± 0.08	40.64 ± 0.07	0.320 ± 0.027
2013/12/07 (3)	407.0 ± 4.0	−8.59 ± 0.05	41.26 ± 0.12	0.325 ± 0.020
a2014/01/07 (2)	N/A	−10.31 ± 0.58	40.31 ± 0.56	0.332 ± 0.001
2014/01/20 (3)	427.5 ± −9.5	−9.53 ± 0.39	40.50 ± 0.21	0.364 ± 0.037

^a^At least one concentration measurement is missing and so standard deviation is not available.
