# Supplementary material for: Oxygen anomaly in near surface carbon dioxide reveals deep stratospheric intrusion
Source: Sci Rep. 2015 Jun 17;5:11352. doi: 10.1038/srep11352 (PMC4469951; doi:10.1038/srep11352)

Supplementary Information

Oxygen anomaly in near surface carbon dioxide reveals deep stratospheric intrusion

Mao-Chang Liang[1,2,3*], Sasadhar Mahata[1]

[1]*Research Center for Environmental Changes, Academia Sinica, Taipei, Taiwan;*

Institute of Astronomy and Astrophysics, Academia Sinica, Taipei, Taiwan

[2]*Graduate Institute of Astronomy, National Central University, Jhongli, Taiwan*

[3]*Department of Physics, University of Houston, Houston, TX, USA*

**To whom correspondence should be addressed: mcl@rcec.sinica.edu.tw*

Table S1: Reproducibility test of $\Delta^{17}O$ in CO_2 using gas from a tank of compressed air with 387.7 ppmv CO_2 and ambient CO_2 collected in duplicates (in 2 1-liter pyrex bottles, connected in series). For duplicate samples, absolute difference in $\Delta^{17}O$ (Diff.) is given. AS-1 CO_2 is a pure CO_2 cylinder, with $\delta^{18}O$=11.53‰ and $\delta^{13}C$=-39.92‰. Values of $\delta^{13}C$ and $\delta^{18}O$ are referenced to V-PDB and V-SMOW, respectively.

387.7 ppmv CO_2 air cylinder					
Sample		$\delta^{13}C$ (‰)	$\delta^{18}O$ (‰)	$\Delta^{17}O$ (‰)	
1		-9.42	25.46	0.207	
2		-9.44	25.45	0.199	
3		-9.40	25.47	0.191	
4		-9.30	25.43	0.210	
5		-9.42	25.44	0.205	
6		-9.45	25.45	0.194	
7		-9.51	25.42	0.196	
8		-9.47	25.45	0.218	
9		-9.49	25.42	0.202	
10		-9.48	25.50	0.209	
11		-9.50	25.26	0.208	
12		-9.50	25.48	0.202	
13		-9.54	25.42	0.202	
14		-9.50	25.49	0.186	
15		-9.52	25.53	0.201	
16		-9.50	25.51	0.203	

17		-9.49	25.47	0.200	
	Average	-9.47	25.45	0.202	
	Stdev	0.06	0.06	0.008	
Ambient air					
	$[CO_2]$ (ppmv)	$\delta^{13}C$ (‰)	$\delta^{18}O$ (‰)	$\Delta^{17}O$ (‰)	Diff. (‰)
Pair #1	423.5	-9.36	40.77	0.374	0.004
		-9.23	40.88	0.378	
Pair #2	420.2	-9.13	41.18	0.372	0.008
		-9.20	41.15	0.380	
Pair #3	417.3	-9.09	41.16	0.406	0.003
		-9.25	41.09	0.409	
				Average	0.005
AS-1 CO_2					
Sample		$\delta^{13}C$ (‰)	$\delta^{18}O$ (‰)	$\Delta^{17}O$ (‰)	
1		-39.92	11.53	0.133	
2				0.145	
3				0.146	
4				0.147	
5				0.131	
6				0.127	
7				0.124	
8				0.155	
9				0.142	
10				0.135	
11				0.146	
12				0.133	

13				0.120	
14				0.132	
15				0.121	
16				0.131	
17				0.137	
			Average	0.136	
			Stdev	0.010	

Table S2: Accuracy for the present experimental setup. Isotope values (in ‰) are referenced to V-SMOW, with primary O_2 and CO_2 from OZTECH (OZTECH Trading Corporation, Safford, AZ, USA). Sample #1-7 are CO_2 made in the laboratory following graphite method to convert O_2 to CO_2. Nominal $\Delta^{17}O(CO_2)$ is either assumed to be the same as initial O_2 used for converting to CO_2 (sample #1-7) or measured by M. Thiemens (sample AS-1). Diff. refers to the difference of the measured $\Delta^{17}O$ from the nominal value. Measured $\Delta^{17}O(CO_2)$ refers to the final value after correction (see ref. 1 for details). Nominal $\Delta^{17}O$ for AS-1 CO_2 (n=1) is done at UCSD, using fluorination system in Thiemens' lab. Note that we do not measure V-SMOW but we do measure air O_2. $\delta^{18}O$, $\delta^{17}O$, and $\Delta^{17}O$ of air O_2 (n=5) are 23.95±0.06‰, 12.07±0.03‰, and -0.213±0.01‰, respectively. For comparison, Barkan and Luz[2] yield 23.881‰, 12.026‰, and -0.223‰. In addition, our primary CO_2 standard is from OZTECH, in good agreement with an independent check using NBS-19 CO_2; the differences in $\delta^{13}C$ and $\delta^{18}O$ between OZTECH CO_2 and NBS-19 CO_2 are about 0.05‰.

		Graphite conversion				
Sample	Nominal $\Delta^{17}O(CO_2)$	$\delta^{17}O(O_2)$	$\delta^{18}O(O_2)$	$\delta^{18}O(CO_2)$	Measured $\Delta^{17}O(CO_2)$	Diff.
1	-0.254	7.708	15.491	15.455	-0.260	-0.006
2	-0.254	7.708	15.491	15.455	-0.257	-0.003
3	-0.254	7.708	15.491	15.455	-0.256	-0.002
4	-0.169	7.762	15.430	15.416	-0.160	0.009
5	0.124	8.044	15.404	15.590	0.132	0.008
6	1.809	12.523	20.827	20.824	1.796	-0.006
7	1.809	12.523	20.827	20.824	1.805	0.003
AS-1	0.15±0.03[a]	N/A	N/A	N/A	0.136±0.010	-0.014

[a]Values were measured in the Thiemens' lab, using fluorination method to convert CO_2 to O_2.

References

1. Mahata, S., Bhattacharya, S. K., Wang, C. H., & Liang, M. C. Oxygen Isotope Exchange between O_2 and CO_2 over Hot Platinum: An Innovative Technique for Measuring $\Delta^{17}O$ in CO_2. *Ana. Chem.* ***85***(14), 6894-6901(2013).
2. Barkan, E., Luz, B. The relationships among the three stable isotopes of oxygen in air, seawater and marine photosynthesis. *Rapid Communications in Mass Spectrometry* ***25***(16), 2367-2369(2011).

Figure S1: Schematic diagram of the vacuum line used in the present experiment to carry out isotope exchange of CO_2 with O_2. The system is designed to handle simultaneously three CO_2 samples. See Table S1 for results showing the reproducibility of the method.

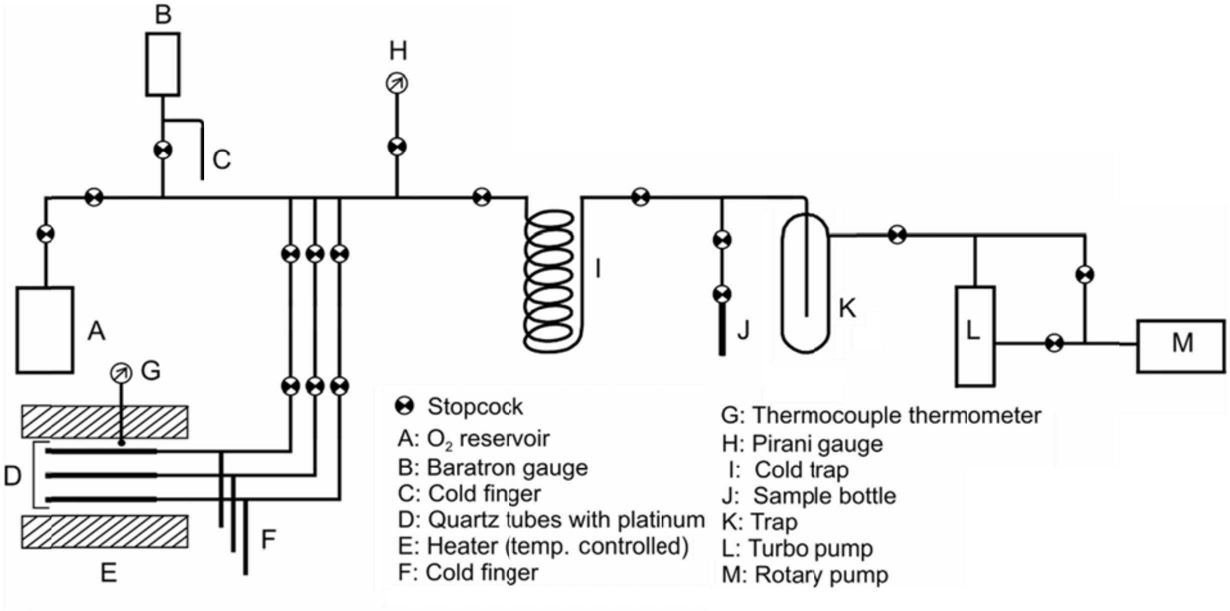

Figure S2: Accuracy of the present experimental setup, taken from Table S2. Black and red circles are for our artificially made CO_2 and AS-1 CO_2 (with 1-σ error bar overplotted), respectively.

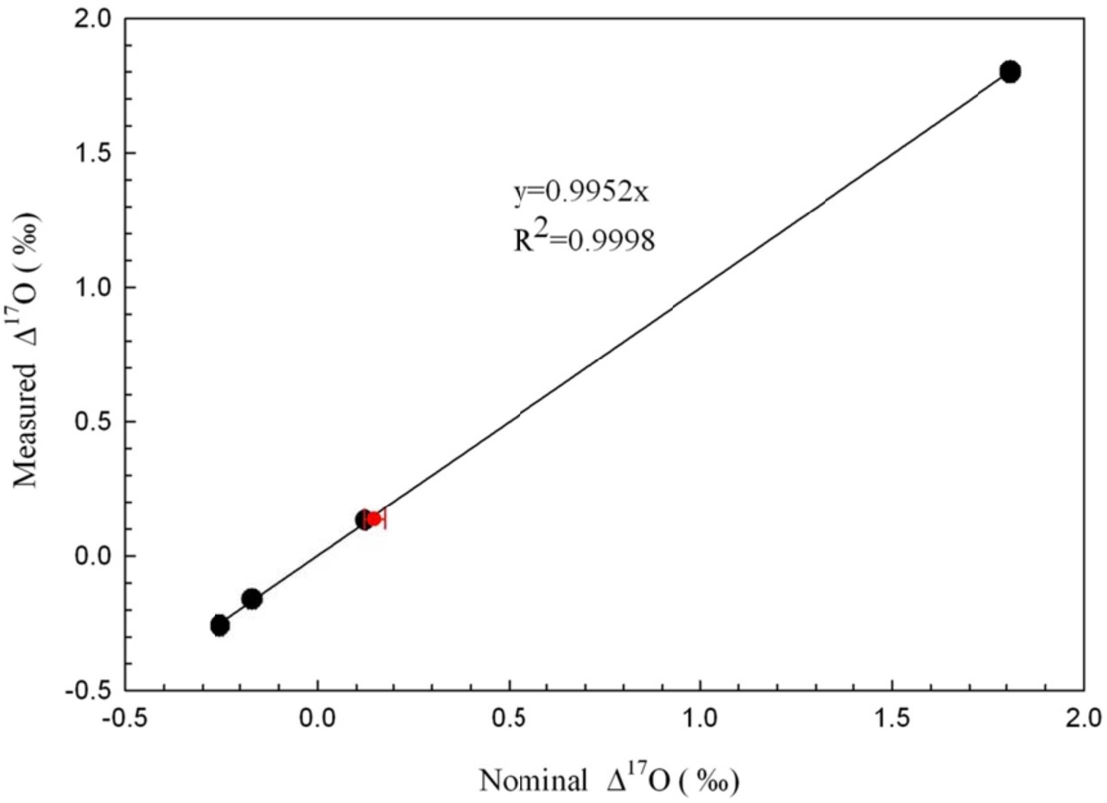

Figure S3: Keeling plots of carbon isotope data for air CO_2 collected. Linear regression line is also given.

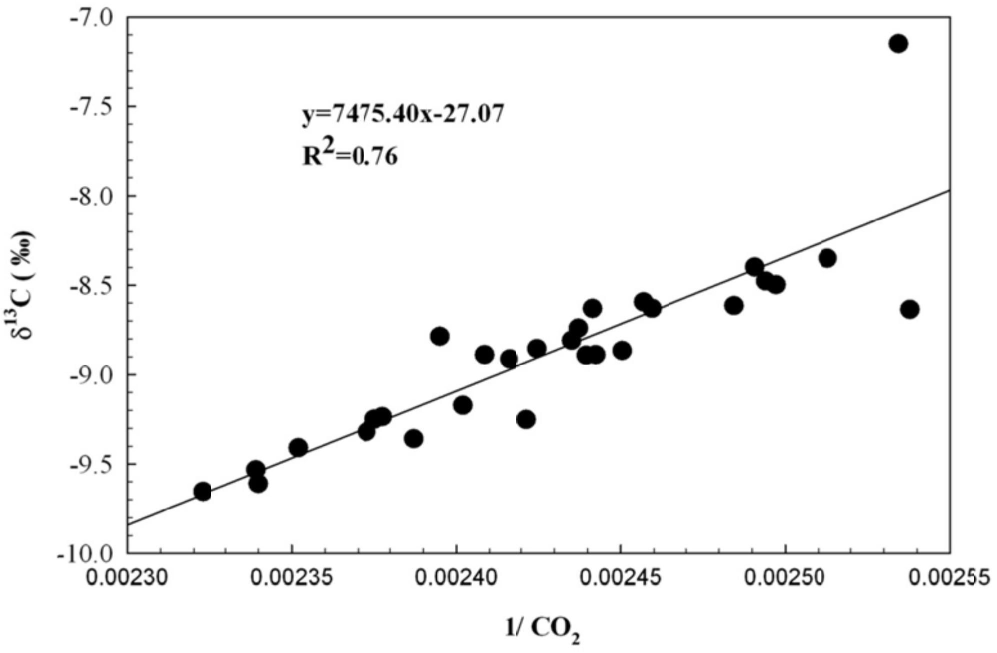

Supplement: Supplementary Information [file srep11352-s1.pdf]
